# Indents

**Published:** 1908-01-15

**Authors:** 


### INDENTS.

☞Brief Articles of Techinical or Scientific Value will receive notice and comment. Contributions invited.

**Setting Crowns with Gutta-Percha.** For setting crowns and bridges, make the following solution:

Chloroform.
Oil of eucalyptus....................aa f ℥ j
Aristol..............................gr. xx
Gutta-percha, q. s.

Make in a wide-mouthed bottle; smear stopper with vaseline to prevent evaporation and make it easy of removal. It will take about a week to make a satisfactory solution, and it should be stirred from time to time, till all lumps have disappeared. Add more gutta-percha if necessary to make a creamy consistency. I prefer the red gutta-percha cut in small pieces. This preparation is to a certain extent anti-septic, due to the mercury sulphide in the gutta-percha and the aristol.—L. G. NOEL, *Dental Cosmos.*

**To Remove a Morbid Growth of Gum Tissue from a Cavity.** Frequently we have cases when a morbid growth of gum tissue fills, or partly fills, a cavity. Its removal is not only somewhat painful, but it is also a mean piece of work to cut it away on account of the excessive hemorrhage. In the majority of such cases it can be removed neatly and with dispatch, painlessly and bloodlessly, by ligating with a piece of silk to either the tooth with the cavity or the adjoining one, whichever is the more convenient. This cuts off the blood supply and reduces to a minimum the pain when cutting away the growth with a side motion of a small, flat burnisher or other suitable instrument. touch with trichloracetic acid. For those who have not in a bad case, tried this method of removing this troublesome growth, the result will be surprising.—*Dental Office and Laboratory.*

**Wire Connection for Lower Partial Dentures.**

A plate of this kind is very much easier to make, less in the way of the tongue, stronger and the life of the remaining teeth will be very much extended by virtue of its use. There can be no criticism made upon the ground of the lack of strength of this wire. The reason is that the wire is drawn through the plate and is of uniform strength throughout its length, whereas in a plate as commonly constructed the swedging or vulcanizing leaves some one point that is weaker than others. At the present time we are using 13 gauge wire for this purpose and in placing it my experience has been that it is better not to fit it too closely to the tissues. Fit it loosely so that it does not press nto the tissues. You may lay it well down in the floor of the mouth, and it will be a great pleasure to those who have not had experience with this form of partial denture to see the results you will get.—F. E. Roach in *Review.*

**Root Bands.**

In this instance the point is not so much whether we believe in a band or not. but is a question of the physiological and mechanical requirements of the crown which the root is to support. These combined demand a union between crown and root which will afford a minimum of irritation and a maximum of strength. If such composite requirements may be obtained to the best advantage without a band, then the use of one is unnecessary, and therefore objectionable; but if the presence of a band will afford a better adaptation of the crown to both the base and periphery of the root, thereby minimizing the possibilities of irritation, and carrying the joint to a more immune area, thus better protecting the mounting medium, which its proper adaptation has usually heretofore afforded, then such a type of construction is not only indicated, but demanded as a practice.

**Sacrificing Natural Crowns.** This question resolves itself into, first, whether a fixed structure would be the best means of supplying the missing teeth or not; and, second, whether an artificial crown would afford the best and most permanent means of obtaining attachment to that tooth. Until the present time an artificial crown has seemed to offer the best means of obtaining such attachment in the most artistic and permanent manner, for the reason that a better adaptation between it and the supporting tooth could be effected, than was so universally possible by any other means at our command. Previous to the successful application of inlay work this was true, because most, if not all, of our former methods were so difficult to adapt with any degree of accuracy that they could only be considered as being of a more or less temporary character, and since a remaining natural crown was thus saved—only to be subsequently lost—such a procedure was often warrantable, and would be so today under the same conditions.

**Evans' Will Contest Settled.** An adjustment of the Evans' estate tangle has been effected after ten years of litigation and affairs are now in shape for the consummation of the famous dentist's plans for a museum in this city. All the papers in the case, including a release signed by the fifty-three contesting heirs, are in the hands of G. Heide Norris, one of the attorneys for the museum society. The adjustment will put the trustees in possession of the dentist's American estate which is valued at close to $1,000,000. It is understood that the former home of Dr. Evan's parents at 40th and Spruce streets will be the site of the "Dr. Thomas W. Evans Museum and Institute." The memorial will fall short of its founder's idea as the estate is worth less than half the valuation placed upon it by Dr. Evans. The heirs get the French estate, which is worth approximately $800,000. Dr. Evans valued his entire estate at about $6,000,000, but there has been a depreciation in property values and litigation ate up a large amount of the funds. It is believed there will be be no more trouble or litigation and the trustees are expected to proceed with the carrying out of the provisions of the will at an early date. The trustees are J. Levering Jones, John Weaver, John H. Converse, Samuel Dickson, W. W. Foulkrod, Samuel B. Reeves, Francis I. Gowen, George W. Norris and Edward Morrell.—*Philadelphia Bulletin.*

**Root-Filling for Deciduous Teeth.** There are a number of methods of varying values recommended for treatment of this condition, none of which, however, fill the root canals with a material which is absorbed with the root, and any other offers a source of irritation for a recurrence of the pathological condition.

To fill the root canals which the osteoclasts are about to absorb to make room for the growing permanent tooth, we must have a material that will be absorbed with it, and will adhere to moist surfaces, as we can not thoroughly dry them; a product which is non-irritating and, if non-medicinal, a conveyor of drugs. For this purpose your essayist wishes to suggest a treatment of formalin and trikresol, equal parts, as recommended by Dr. J. P. Buckley, for sterilization, which is to be removed in forty-eight hours. After the removal of carious matter the root canals are filled with this formula:

| | |
|---|---|
| R.—Isinglass | dr. i |
| Tannic acid | gr. iss |
| Trikresol | m. iv |
| Aqua dist | ir jss |

This when heated to a temperature of 100° in an ordinary gluepot or water bath becomes syrupy and can be readily introduced into the root canals with a piece of sterile catgut. If the canal be large the catgut may be left in the canal. A ball of stiff phosphate of zinc is then pressed into the pulp chamber forcing the mixture through the canal and sinus. The cavity is then filled and contoured with amalgam.—H. C. Ferris, in *Items of Interest.*

**Fixed and Removable Bridge-Work.** If the position and stability of the teeth which remain, and which may be used to support the structure supplying the missing teeth, are favorable and adequate to the mechanical or dynamic requirements of a fixed structure, then such a type of construction is indicated, but in all cases where this may be at all doubtful, then a "removable" piece is demanded. Hence the success of the procedure will depend not so much upon the selection made from the vast array of methods at our command, but, on the contrary, must rest more or less entirely upon the appreciation of mechanics exercised by the operator. Indeed, my sympathy goes out to him in whom this faculty is not developed, and to his patients also when he essays to do dental bridge work. Such an analysis of these so-called problems leads us to the conclusion that they are not questions of principles, but rather of judgment. Therefore, it behooves us to cultivate and develop this attribute to a higher degree if we would hope to aid in placing this specialty on a broader scientific and less empirical plane. If this degree of judgment prevails, first in the application of correct principles, and, second, in the selection of methods of procedure, let me again prophesy that we will find ourselves discarding old methods, if indeed we have not already done so, and using even a lesser number of the new ones, and the practice of crown and bridge work will therefore become practically revolutionized.—H. J. Goslee in *Items of Interest.*

**Personal Appearance.** Let us begin the day with this money-making dentist. He arises at seven o'clock; one-half hour is devoted to his toilet; a clean shave —no dentist who does not wear a beard has the right to present himself unshaven to a patient; a cold sponge and a good rub. His teeth and hair are given proper attention, he dresses with fresh linen, and a well tailored business suit. Clothes do not make the man, but they play a mighty important part when the world takes a look to size you up.

Shabby clothes are no longer an allowable eccentricity of genius. Good clothes are a good investment. The dentist's neckwear is admired or criticized by the ladies more than we know. One two-dollar tie is a better investment than four at fifty cents. He is now well dressed. This one thing is a step to the large fees we find in his practice. He next looks over the morning paper. At eight he has finished a light breakfast. A brisk walk, and he arrives at his office ready and fit for a stiff day's work. At his office he spends ten minutes to clean his teeth, manicure his nails, and douche his nose and throat. He considers his nose and throat require as much attention as his teeth. Stop and think that the patient looks directly into the nose when you are operating. He is sure his breath is not offensive. A deodorant is used before the attendance upon a patient. After getting into a clean operating coat, the morning mail is looked over, and the answers dictated to the lady assistant. At nine he meets his appointment promptly on time. The patient is placed in the chair and made ready for operating by the lady assistant. The dentist steps to the chair and greets the patient with a cheerful good morning and a smile. You can not set a better example to your patients than to meet your appointment on time. Many a dentist has lost good patients by not being on time.—*Western Journal.*

**Crown and Bridge-Work of the Future.** Again, venturing a prophesy for the future, let me suggest what I think will be the composite of typical and ideal methods.

For single crowns the all porcelain, hollow, or "jacket" crown is undoubtedly one of the most practical and cosmetic means of restoring the anterior teeth, and while it will probably come into more general use than it is at present, still the high order of skill required, and the fact that such a type of construction is not universally applicable, will necessarily limit the field of its usefulness.

The use of replaceable porcelain teeth without platinum pins, to be subsequently attached by cement, for individual crowns and also for dummies, or substitutesfor the natural eeth in bridge work, must be considered as the solution of the problem of discolored and fractured facings, for the reasons mentioned, and hence is undoubtedly destined to become the practice of the future as soon as we can prevail upon the manufacturers to supply our wants and needs in this direction.

With porcelain teeth suitable fòr this purpose—and we will get them some day—we will thus have two general types of construction for single crowns, types which will embrace a field more or less universal in application and general usefulness, for all teeth within the range of vision. Combine these with the ordinary gold shell crown made to fit and to occlude properly, and applied to teeth so removed from the range of vision as to eliminate any objections from a cosmetic view-point, and we find a limited number of types, with an almost unlimited range of application.

Having also one general type of dummy for bridge work which will be equally practical, esthetic and applicable in the construction of dental bridges, then we will need but to consider what shall be the type of attachment to the supporting teeth, and I am of the opinion that three general types will ultimately answer our purposes in a very large majority of cases. The replaceable porcelain crown with cast base for anterior roots, where the substitution of the entire crown is indicated; the gold telescope crown for posterior roots, where crowning is demanded, and the inlay where all or even a sufficient portion of the crown of the natural tooth remains, and these attachments are equally applicable to "removable|" as well as to "fixed" structures.

Thus may the construction of crown and bridge work be revolutionized, and, therefore, since we have these splendid possibilities ahead of us, must its practice become less empirical and more systematic, practicable, cosmetic, and successful.—H. J. Goslee, jn *Items of Interest.*

**Cast Inlays.** In this operation there are five distinct steps: First, preparing the cavity. Second, carving the wax model. Third, investing this model in the flask. Fourth, causing the wax model to disappear and casting the inlay. Fifth, finishing the inlay and cementing it in the cavity.

In the preparation of the cavity there are four points to consider: First, such form as will permit the withdrawal of the wax without distortion. Second, such form of the gingival or pulpal walls as will provide so firm a *seat* for the filling that it will not rock on its base. Third, such retention form as will provide resistance against such stresses as would tend to *unseat* the filling. Fourth, such form of enamel margins as will protect the enamel from fracture and give such marginal form to the inlay as will prevent change under stress. (Proximal cavities in bicuspids and molars are the ones considered here.)

In the actual preparation of the cavity the first two steps are practically one, and in producing the conditions mentioned we usually find that we *have* made the gingival and pulpal walls flat mesio-distally at least and parallel with the horizontal plane of the tooth, the axial walls rising from them at *definite* angles parallel with the vertical axis of the tooth. Those operators who prepare their cavities for metallic fillings in accord with the principles enunciated by Dr. Black will find little difficulty in adapting their cavity preparations to the requirements of the inlay. In fact, some of my specimens were originally prepared for cohesive gold fillings, the only change being the elimination of the linguo-gingival and bucco-gingival retention and the straightening of the walls of the step so the wax would *draw*.

In providing retention against stress we must consider the direction and amount of the stress and the form of the occlusal surface of the completed inlay. In bicuspids and molars stress is delivered upon the occlusal surface within a radius of from twelve to twenty centigrades with the vertical axis of the tooth. In accordance with dynamic laws, resistance to such stress should be provided as near the point of delivery of the stress as possible—in these cases it should be in the occlusal third of the cavity.

If the occlusal surface is not involved so that a step is not employed the buccal and lingual walls should join the axial wall at such an angle as to give a dovetail, with its base formed by the axial wall, especially in the occlusal third. If a step is employed the retention may be entirely in the step and should show a dovetail form as we look down upon the occlusal surface. The *amount* of retention necessary is governed by the *amount* of stress. In a perfect inlay operation the cavity preparation should be such that the inlay will remain firmly seated, *without cementing*, against any stress excepting that the necessity to withdraw it in a line parallel with the vertical axis of the tooth.

In the preparation of enamel margins the same care should be exercised and the same laws observed as in cohesive gold fillings. The greater density and strength of the cast filling may, however, permit of greater bevels of the margins of the inlay. The greatest case should be exercised that all lines be *true* and all margins definite and smooth. In the preparation of cavities Dr. Taggart advises the use of small *true* carborundum discs and points as giving a better preparation and being more humane, as they can and *should* be kept wet by continuous stream of water. Each part of the cavity should have a stone shaped especially for it, which should be used nowhere else.— Thos. Weeks in *Brief*.

